# Japanese Encephalitis Virus wild strain infection suppresses dendritic cells maturation and function, and causes the expansion of regulatory T cells

**DOI:** 10.1186/1743-422X-8-39

**Published:** 2011-01-26

**Authors:** Shengbo Cao, Yaoming Li, Jing Ye, Xiaohong Yang, Long Chen, Xueqin Liu, Huanchun Chen

**Affiliations:** 1 State Key Laboratory of Agricultural Microbiology, Huazhong Agricultural University, Wuhan, Hubei 430070, PR China; 2 Laboratory of Animal Virology, College of Veterinary Medicine, Huazhong Agricultural University, Wuhan, Hubei 430070, PR China; 3 College of fisheries, Huazhong Agricultural University, Wuhan, Hubei 430070, PR China

## Abstract

**Background:**

Japanese encephalitis (JE) caused by Japanese encephalitis virus (JEV) accounts for acute illness and death. However, few studies have been conducted to unveil the potential pathogenesis mechanism of JEV. Dendritic cells (DCs) are the most prominent antigen-presenting cells (APCs) which induce dual humoral and cellular responses. Thus, the investigation of the interaction between JEV and DCs may be helpful for resolving the mechanism of viral escape from immune surveillance and JE pathogenesis.

**Results:**

We examined the alterations of phenotype and function of DCs including bone marrow-derived DCs (bmDCs) *in vitro *and spleen-derived DCs (spDCs) *in vivo *due to JEV P3 wild strain infection. Our results showed that JEV P3 infected DCs *in vitro *and *in vivo*. The viral infection inhibited the expression of cell maturation surface markers (CD40, CD80 and CD83) and MHCⅠ, and impaired the ability of P3-infected DCs for activating allogeneic naïve T cells. In addition, P3 infection suppressed the expression of interferon (IFN)-α and tumor necrosis factor (TNF)-α but enhanced the production of chemokine (C-C motif) ligand 2 (CCL2) and interleukin (IL)-10 of DCs. The infected DCs expanded the population of CD4+ Foxp3+ regulatory T cell (Treg).

**Conclusion:**

JEV P3 infection of DCs impaired cell maturation and T cell activation, modulated cytokine productions and expanded regulatory T cells, suggesting a possible mechanism of JE development.

## Background

JEV is a causative agent of JE which causes at least 50,000 clinical cases and about 10,000 deaths each year. It is a member of the mosquito-borne encephalitis complex of the *Flaviviridae *family and has recently been discovered in previously non-affected areas like Australia [1] and Pakistan [2]. The neurons in the central nervous system (CNS) are target cells of JEV. Studies show that a direct viral cytopathic response and both direct and indirect immunological responses can contribute to CNS degeneration through JEV-infected cell exclusion by macrophages and CTLs, secretion of cytokines and chemokines and activation of microglia [3-6]. However, few studies have investigated the mechanisms by which JEV evades the immune surveillance of the host and passes through the blood-brain barrier (BBB) to the CNS.

Dendritic cells (DCs) are the most prominent antigen-presenting cells (APCs) which induce dual humoral and cellular responses. While DCs also play unique role in inducing immune tolerance, avoiding immune surveillance and causing persistent infection. There are studies about the interaction between virus and DCs which showed that viral infection of DCs inhibited the cell maturation and impaired the cell function [7-9]. Human cytomegalovirus (HCMV) infection de-regulated the expression of surface MHC classⅠ, CD40, CD80 and CD86 molecules on DCs. Furthermore, both T cell proliferation and cytotoxicity of T cells specific to an antigen presented by DCs were reduced via the release of soluble CD83 when DCs were infected with HCMV [8,10,11]. Likewise, human immunodeficiency virus (HIV) affected maturation of DCs within the thymus, which contributed to the loss of the naive T cell and memory T cell population and even facilitated the dissemination of HIV [12].

Additionally, recent studies revealed that several viruses belonging to the *Flaviviridae *family, such as classical swine fever virus (CSFV), Dengue virus (DV) and Yellow fever virus (YFV), infected DCs and altered the cell phenotype and function [13-15]. Furthermore, Aleyas et al. [2009] recently reported that JEV Beijing strain replicated both in bmDCs and macrophages, and induced functional impairment of DCs through MyD88-dependent and independent pathways which subsequently led to poor CD4+ and CD8+ T cell responses [16]. Thus, the investigation of the interaction between virus and DCs is imperative for resolving the viral escape from immune surveillance and JE pathogenesis.

Since there is no evidence for JEV infection of DCs *in vivo*, we investigated the alteration of phenotype and function of the JEV P3-infected DCs both *in vitro *and *in vivo*. Our results indicated that JEV P3 severely infected DCs *in vitro *and *in vivo*, and the infection with JEV impaired cell maturation and the capacity for T cell activation. In addition, our study also showed that the infection of DCs with P3 expanded the population of CD4+ Foxp3+ regulatory T cell (Treg) with immunosuppressive potential, suggesting that the virus-induced alteration of DCs is a likely cause of the immunosuppression found in JEV infection.

## Results

### JEV P3 infection of DCs *in vitro *and *in vivo*

**Figure 1:**
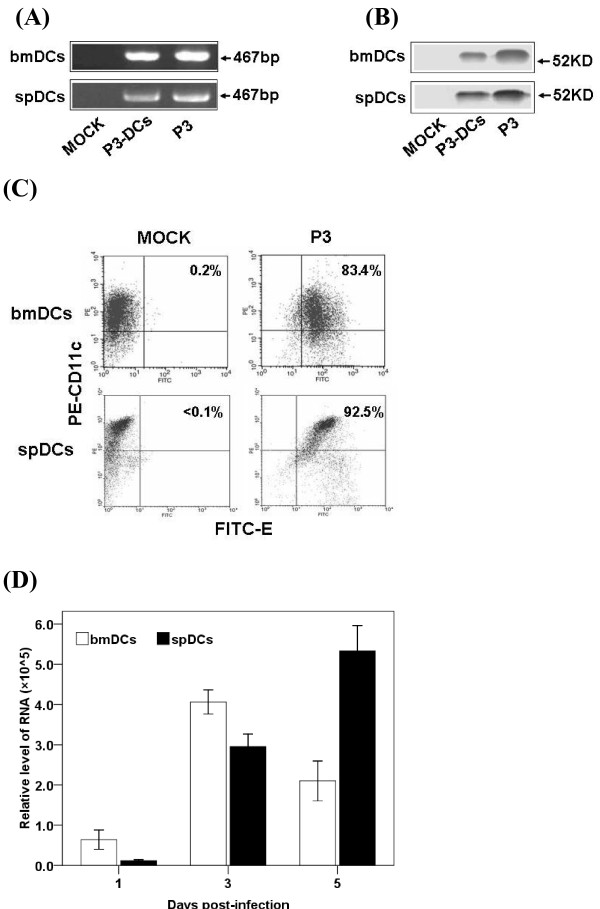
**P3 infects DCs *in vitro *and *in vivo***. (A) The *in vitro *infected bmDCs and the spDCs from P3-challenged mice were harvested and analyzed with RT-PCR. Bands shown are 467-bp PCR products specific for JEV. (B) The bmDCs and spDCs were analyzed for E protein (JEV envelope protein) by separation of the proteins on a 10% SDS-PAGE gel followed by electrotransfer to NC membranes and incubation with monoclonal antibodies against E protein. (C) The bmDCs were harvested after 3 days infection and the spDCs were isolated from mice which had been challenged for 5 days. 1 × 10^5 ^bmDCs or spDCs were doubly stained with FITC-anti-E and PE-anti-CD11c and analyzed by FACS respectively. (D) The infected bmDCs and the spDCs from challenged mice were collected 3 times at day 1, 3 and 5, and a real-time PCR was performed to quantitatively detect RNA copies of JEV. Each point represents the mean ± SD determinants in triplicate.

The purity of the bmDCs fraction from cell culture or infected mouse splenocytes was higher than 90% as determined by FACS analysis with surface molecules expression (CD11c). After JEV infection, a 467-bp specific RNA fragment of JEV was detected by RT-PCR (Figure 1A) and the E protein of the JEV was detected by Western blotting in DCs (Figure 1B). FACS results showed over 80% bmDCs and 90% spDCs were infected by JEV P3 (Figure 1C). Analysis by real-time PCR showed that DCs supported JEV replication and yielded infectious virus (Figure 1D). These results suggest that JEV infected DCs both *in vitro *and *in vivo*.

### P3 infection suppressed the maturation of DCs

**Figure 2:**
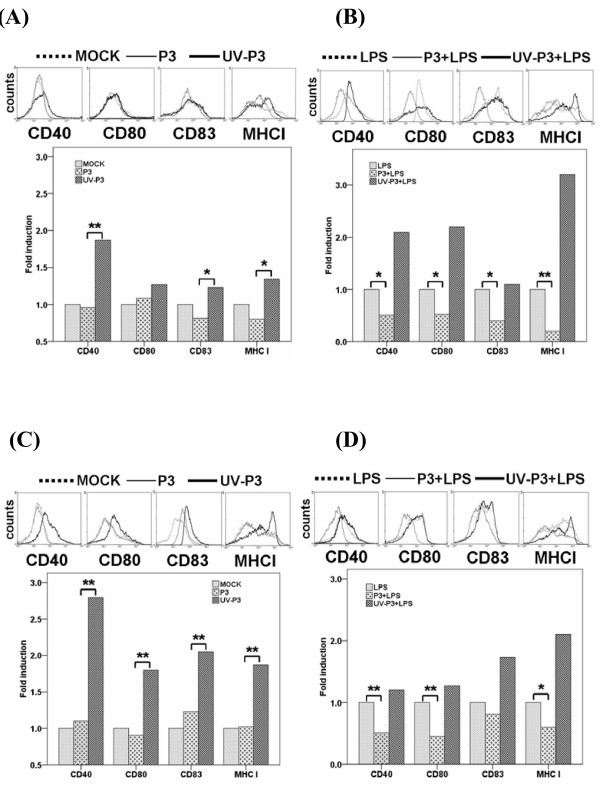
**Effects of P3 infection on DCs maturation**. 1 × 10^5 ^freshly purified bmDCs were left mock-treated or treated with 1 MOI of P3 or UV-P3 with or without LPS (lipopolysacchide, Sigma-Aldrich, MO) for 3 days. The spDCs from mice, which have been challenged or immunized for 5 days, were obtained and treated with or without LPS. Expressions of CD40, CD80, CD83 and MHCⅠ of the bmDCs (A,B) or spDCs (C,D) were evaluated by FACS. Relative fluorescence intensity to mock group (fold induction) was expressed as the means ± SD of triplicates. *, *P *< 0.05; **, *P *< 0.01.

DCs present antigen to and activate T lymphocytes through up-regulating the expression of costimulatory and antigen presentation-associtated molecules at the mature stage [17]. To examine whether the characteristics of immature DCs were altered by P3 infection, we tested the surface molecules of the infected DCs *in vitro *and *in vivo*. The expression of maturation surface markers, including CD40, CD80, CD83 and MHCⅠwas up-regulated in UV-P3-stimulated, but not in P3-infected bmDCs and spDCs or mock-treated DCs (Figure 2), indicating that UV-P3 stimulation accelerated the maturity of DCs whereas P3 infection dramatically inhibited the cell maturation process.

### P3 infection modulated cytokine production of DCs

**Figure 3:**
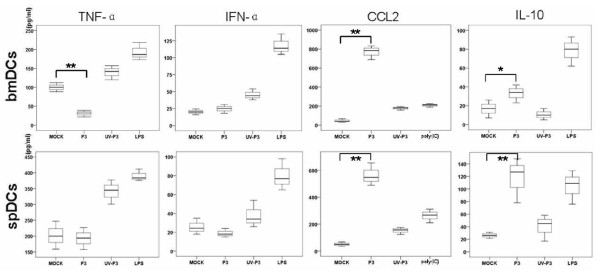
**Cytokine profiles of P3-infected DCs (IFN-α, TNF-α, CCL2 and IL-10)**. 1 × 10^5 ^freshly purified bmDCs were left mock-treated or treated with 1 MOI of P3 or UV-P3 for 3 days. The spDCs from mice, which were challenged or immunized for 5 days, were obtained and cultured for 3 days. The cell supernatants harvested at 3 days of post infection were analyzed with ELISA to measure the concentrations of cytokines (IFN-α, TNF-α, CCL2 and IL-10). Cytokine concentrations were expressed as the means ± SD of triplicates. *, *P *< 0.05; **, *P *< 0.01.

In many cases, virus does not directly result in the destruction of host organism but instead causes indirect damage through the disordered release of cytokines [18]. In addition, imbalanced levels of cytokines may contribute to viral persistence and irreversible immunsuppression. Therefore, we examined the profiles of pro- and anti-inflammatory cytokines produced by P3-infected DCs *in vitro *and *in vivo*. Our results showed that P3 infection enhanced the releases of IL-10 and CCL2 of DCs but suppressed the production of IFN-α and TNF-α (Figure 3). And it was interesting to show that JEV which was inactivated by UV irradiation failed to induce the production of IL-10 and CCL2 but succeeded in inducing the expression of IFN-α and TNF-α. This indicates that the release of CCL2 and IL-10 from DCs was dependent on viral replication, while the production of IFN-α and TNF-α was independent on viral replication.

### DCs infected with P3 attenuated allostimulatory activities to T cells

**Figure 4:**
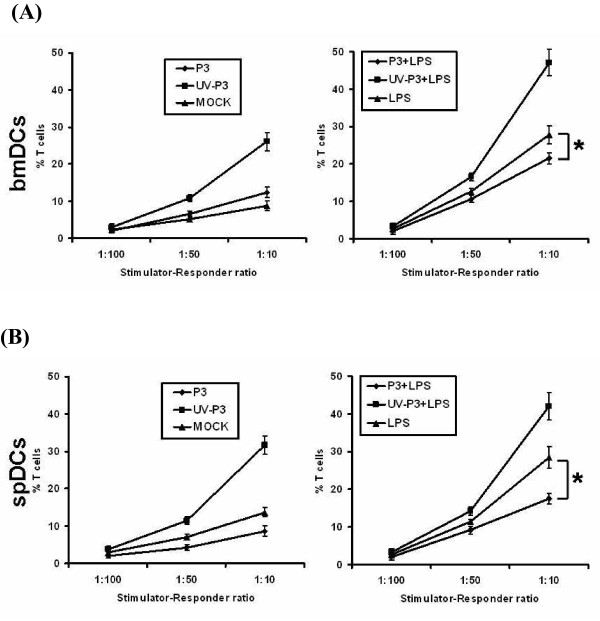
**Effects of P3 infection on DCs activation of naïve T cells by MLR**. Mock-treated, P3-infected or UV-P3-stimulated DCs as well as differently treated spDCs were added in grade dose to 1 × 10^5 ^allogeneic T cells at the indicated stimulator-responder ratios in triplicate, with (B) or without (A) LPS treatment for 20 h before the addition of 50 μl of CellTiter 96^® ^AQ_ueous _One Solution Cell Proliferation Assay. The bmDCs, spDCs as well as T cells were served as spontaneous NADH/NADPH releases controls respectively. The presentation activities of differently treated bmDCs were measured as 100% (OD490_DC+T exp._-OD490_DC spont._-OD490_T spont._)/(OD490_T spont._). Results were expressed as the means ± SD of triplicates. *, *P *< 0.05.

To test whether P3 infection will impair the ability of DCs to activate allogeneic naïve T cells, the direct effect of P3-infected DCs in activation of naïve T cells was analyzed by mixed lymphocyte reaction (MLR) and ELISPOT assay. In MLR, the allo-stimulative capability of DCs was significantly suppressed by P3 infection compared to the UV-P3-stimulated group (*P *< 0.05). In addition, the viral infection blocked the LPS-induced allostimulatory activity of DCs (Figure 4A, B).

**Figure 5:**
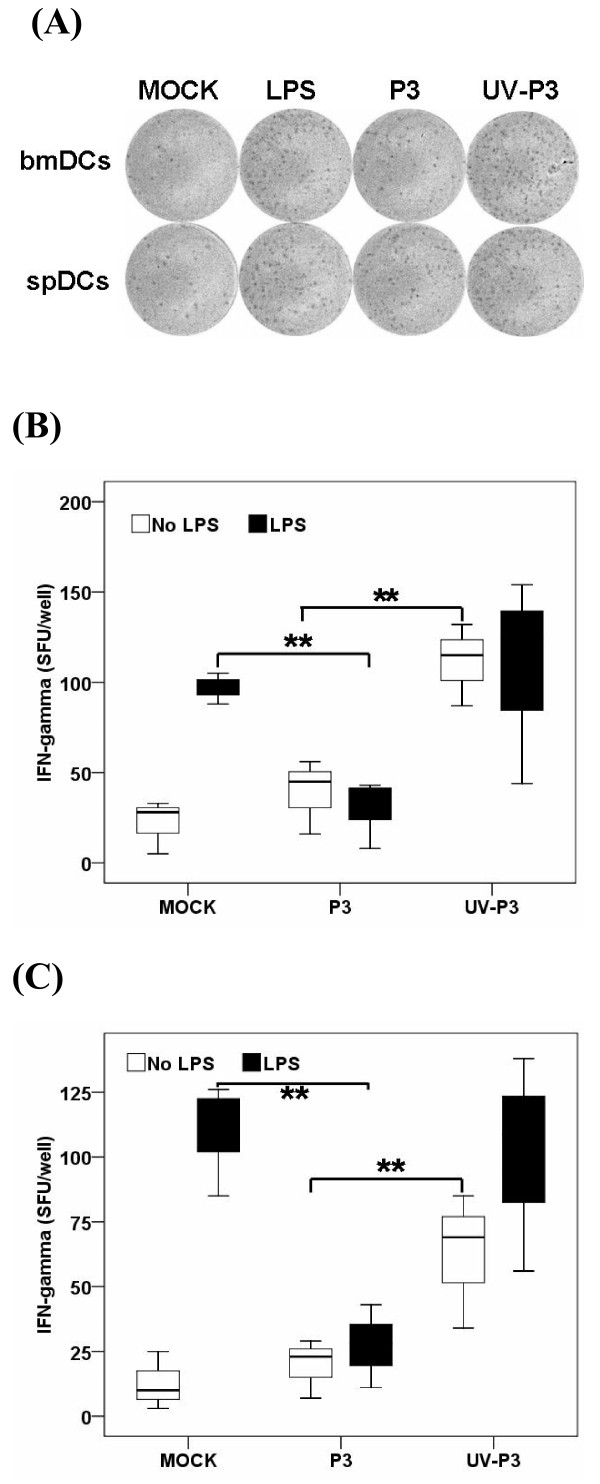
**IFN-γ producing T cells were detected by ELISPOT assay**. P3-infected, UV-P3-stimulated or mock-treated DCs as well as differently treated spDCs were harvested and treated with Mitomycin C (Sigma-Aldrich, MO) at final concentration of 10 μg/ml for 1 h. The differently treated or mock DCs were seeded (1 × 10^4 ^per well) together with 1 × 10^5 ^per well T cells in triplicates for 20 h. LPS-stimulated DC/T cell co-cultures served as positive controls. One representative for IFN-γ spot forming unit (SFU) by ELISPOT assay was shown (A). The figure was representative of three independent experiments. Corrected data (SFU)/well were shown for bmDCs and spDCs activations for naïve T cells to expand and produce IFN-γ by ELISPOT assay (B, *in vitro*; C, *in vivo*). Results were expressed as the means ± SD of triplicates. *, *P *< 0.05.

In ELISPOT assay detecting IFN-γ producing T cells, the number of spot forming units/10^6 ^purified T cells was counted after twenty four hour incubation with differently treated bmDCs or spDCs. The results in vitro showed that P3-infected bmDCs activated 25 ± 9 spots/10^6^, while the UV-P3-stimulated bmDCs activated 68 ± 21 naïve T cells/10^6^. In vivo, P3-infected spDCs produced 52 ±12 spots/10^6 ^whereas UV-P3-stimulated spDCs produced 107 ± 34 spots/10^6^. This was consistent with the result of MLR assay. P3 infection, *in vivo *or *in vitro*, significantly suppressed the ability of DCs to activate allogeneic naïve T cells in response to LPS treatment (Figure 5A, B and 5C). It implied that P3 infection played an important role in the dysfunction of DCs in activating allogeneic T cells.

### P3-infected DCs expanded Treg

**Figure 6:**
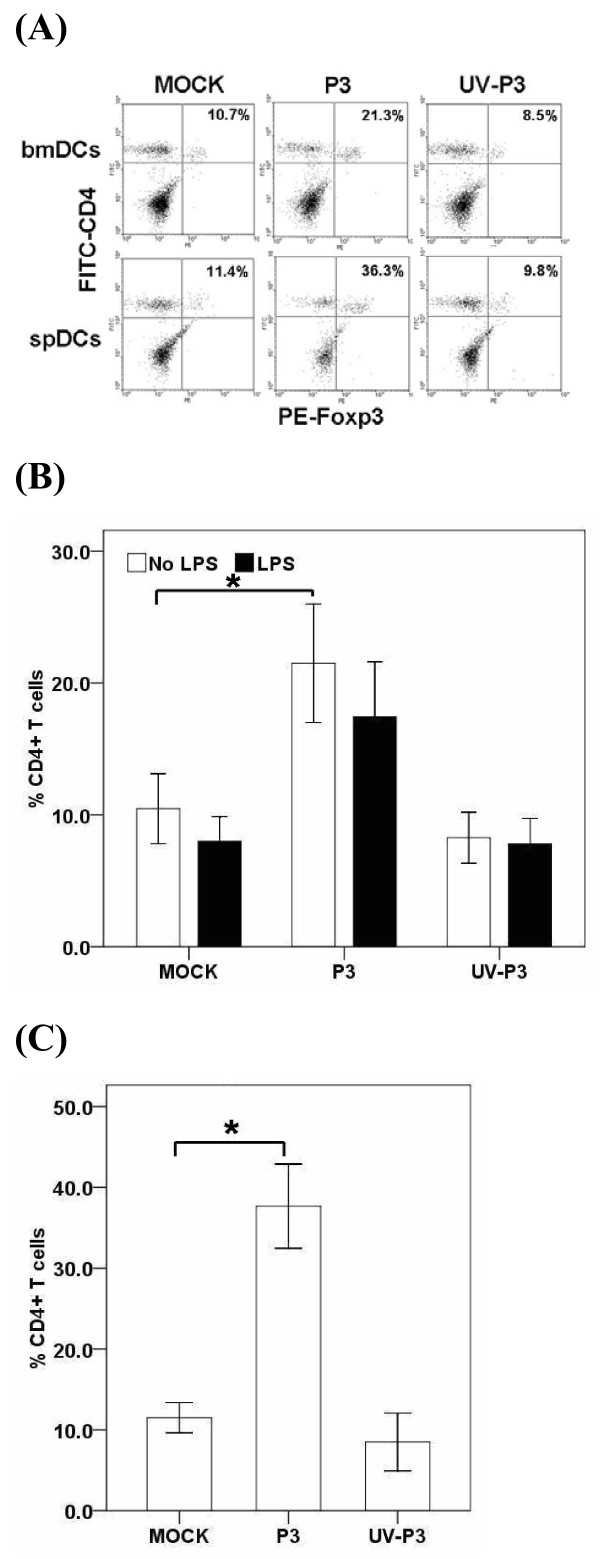
**Effects of P3 infection on DCs-induced differentiation of regulatory T cells**. 1 × 10^5 ^mock-, P3-, UV-P3- or LPS-treated bmDCs were incubated with 1 × 10^6 ^allogeneic naïve T cells for 5 days. T cells were purified and doubly labeled for CD4 and Foxp3, and assessed by FACS. The *in vivo *Treg in splenocytes were purified and examined by FACS from mice inoculated with 1 × 10^5 ^PFU P3 or identical UV-P3 i.p. for 5 days. Representative result was shown from three independent experiments (A). The percentage represented the ratio of CD4+ Foxp3+ cells in CD4+ T cells. P3-infected bmDCs elicited the Treg differentiation *in vitro *(B). After P3 infection or UV-P3 stimulation of mice i.p., Treg differentiation *in vivo *was analyzed immediately (C). Results were expressed as the means ± SD of triplicates. *, *P *< 0.05.

The immune response may be limited in magnitude and efficacy when the host with normal Treg function is infected with virus. We examined whether P3-infected DCs would modulate Treg differentiation. The test revealed that P3-infected bmDCs significantly enhanced the differentiation of Foxp3+ Treg *in vitro *which was consistent with the results *in vivo *(Figure 6A, B and 6C). However, the UV-P3-stimulated DCs did not alter the expansion of the Treg, as well as the mock-treated DCs.

## Discussion

Most studies conducted to evaluate the pathogenesis of JEV infection have noted the interaction of the virus with macrophages, microglia and astrocytes, which are major contributors to the production of inflammatory cytokines and CNS degeneration [3,4,6]. In the present study, we attempted to address the possible pathogenesis of JEV wild strain infection by testing the interaction of JEV and DCs *in vivo *and *in vitro*.

Carrasco et al., [2004] discovered that CSFV could infect and replicate in monocyte and myeloid-derived DCs [14]. Therefore, we hypothesized that JEV, which also belongs to the *Flaviviridae *family, may affect DCs to facilitate viral spread by escaping immune surveillance. Although Aleyas et al. [2009] recently reported JEV infection of DCs *in vitro*, whether JEV infects DCs *in vivo *remained unknown until now. Our research not only verified the results of Aleyas [16], but also investigated the JEV infection of DCs *in vivo*. Additionally, one of our preliminary experiments showed that when BALB/c mice were inoculated with C6FeK4N6-labeled P3-infected bmDCs or spDCs via intraperitoneal (i.p.), JEV and C6FeK4N6-labeled DCs were detected simultaneouly in the brain of mice with severe symptoms of immunohistochemistry (unpublished data). It is likely that JEV could use DCs as a virus delivery vehicle as it moves through the CNS.

The impaired surface molecule expression of APCs may directly affect the process of antigen presentation and T cell activation. Thus, we analyzed the alteration of the surface-molecule expression of infected DCs *in vitro *and *in vivo*. The FACS analyses revealed an suppressed expression of surface molecules, such as CD40, CD80, CD83 and MHCI, on P3-infected DCs *in vitro *and *in vivo*, which is in accordance with Aleyas's results [16]. While we also discovered that the antigen presenting-associated molecules on bmDCs were significantly enhanced after JEV SA14-14-2 strain (a successful JEV live vaccine strain) infection [19]. This suggests the potential molecular mechanism of the immune escape of P3 and the high immunopotency of SA14-14-2.

Since we have verified that JEV infection impaired the expression of antigen presenting-molecules and co-stimulator molecules, whether this impairment of the crucial components on DCs would affect their capacity to activate CD4+ and CD8+ T cell directly is needed to be investigated[20,21]. Thus, we analyzed the capacity of the infected DCs for activating allogeneic T cells by MLR and ELISPOT assay. It was observed that the T cell activating ability of was dramatically impaired by P3-infection, but boosted by UV-P3 stimulation and SA14-14-2 infection. It has been reported that Hepatitis C virus (HCV), Ebola viruses and HIV escaped immune surveillance during acute or chronic infection because of the defect of APCs function for activating T cell [21-23]. Therefore it suggested that the impairment of activating of allogeneic naïve T cells of P3 infected DCs could be involved in the JE development.

Treg is a subset of CD4+ T-cell with regulatory properties. Previous studies on the role of Tregs in viral infections suggest that they suppresses antiviral effector T cell responses or local immune activation at the sites of viral replication [24,25], which may subsequently result in viral immune evasion and the establishment of chronic infections [26-28]. Our FACS results showed that P3 infection contributed to the differentiation of Treg *in vivo*. The results also demonstrated the expansion of Treg population after the co-culture of P3-infected DCs and T cells. It suggested that JEV infection of DCs might influence the mode of T-cell differentiation. Thus, we assumed that induction and expansion of Treg cells by JEV-infected DCs may be associated with immunosuppression in JEV infection. It has previously been shown that immature DCs induced Treg cells are able to suppress other T-cell responses [29-33]. Furthermore, it has been demonstrated that the increased production of IL-10 played an important role in Treg responses which appeared to contribute to immune dysfunction, accounting for viral persistence and acute tissue damage. Therefore, the up-regulation of IL-10 in P3-infected DCs may partly contribute to the expansion of Treg. Based on these results, we suggest that P3 infection may have led to the expansion of Treg cell population *in vivo*, which could have been involved in the suppression of anti-JEV immune responses. In addition, it is essential to note that although CD25 is expressed on most regulatory T cells, it is not specific since it can also be expressed on activated CD4+ T cells [34,35]. Foxp3 has been shown to be a better marker for CD4+ CD25+ T regulatory cells.

The key cytokines secreted by DCs, including typeⅠIFN (IFN-α/β), TNF-α, IL-10 and CCL2, restrict the proliferation of invading pathogens and determine the polarization of Th1 and Th2 [36-38]. In particular, secretion of type I IFN is a key step in the innate immune response to viral infection and TNF-α released by DCs can further recruit DC precursors and sustain the antigen presentation [22]. The impaired expression of IFN-α and TNF-α of DCs following the JEV P3 infection when compared with UV-P3 was observed in the present study may contribute to the attenuated generation of antiviral immune response of the host. However, the report of Chang et al., [2005] revealed JEV infection induced IFN-β participated in fighting the invading pathogens by using cell types of A549 and SK-N-SH cells through IRF-3- and NF-κB-mediated pathway [39]. Similar results were also obtained in the studies of West nile virus (WNV) infection which induced the IFN-α production of pDCs and mDCs [40], while inhibited the IFN-β expression of Hela cell [41]. Therefore, we hypothesized that the different cell types from different tissues may present distinct immune response against viral infection. It is known that different cell types usually exert different functions. For instance, pDCs, which generate the crucial signal adaptor IRF7, constitutively express IFN-I. On the contrary, the expression of IFN-I is extremely inhibited in those cell types in absence of the receptor TLR7/TLR9 and IRF-7 [42,43]. Furthermore, different types of cytokines are usually used to discriminate the patterns of immune responses. Therefore, when only considering the individual cell type, different cell types may present distinct immune responses.

TNF-α level in serum and cerebrospinal fluid (CSF) of the fatal case in significantly correlated with prognostic outcome in wild type JEV infection [44]. Therefore, TNF-α may play an important role in immunopathogical responses of the infected host. However, JEV infection of DCs reduced the expression of TNF-α in the current study. On one hand, it usually appears of appropriate expression of TNF-α from the innate response of the host when external pathogen invading. On the other hand, the excess TNF-α induced cell degeneration could be harmful to the survival of virus itself. Therefore, we speculate that the wild type virus may evolve a mechanism by which to restrict the excess inflammatory factors expression at the beginning of the infection, which may facilitate the persistence of the virus survival. Moreover, P3 infection significantly enhanced the release of CCL2 and IL-10. The IL-10 is considered as an anti-inflammatory factor and plays an important role in the differentiation of Treg cells [31,45,46]. The suppressed TNF-α production in P3-infected DCs may be partially regulated by high-expressed IL-10. Our results indicated that the release of CCL2 and IL-10 from DCs was positively related to viral infection while the production of IFN-α and TNF-α was negatively related to viral replication. We speculate that the temporary presence of some non-structure proteins or dsRNA of JEV during the viral replication may play an important role in decelerating or accelerating certain signaling pathway.

Additionally, most data obtained in our experiments are consistent with Aleyas's results except for decreased production of TNF-α. This contradicted finding about decreased production of TNF-α might be due to various factors, such as the DCs purity (>90% vs >75%), JEV strain (P3 and Beijing) and MOI values. All together, the increased level of IL-10 and the decreased productions of IFN-α and TNF-α presented an immune-suppressive profile, indicating the process of the fatal JE development.

## Conclusion

Our data reveals that JEV P3 could infect mouse DCs in vitro and in vivo, and the infection affects the phenotype and function of DCs, including reducing expression of costimulatory molecules, modulating secretion of crucial cytokines, suppressing activation of T cells, and stimulating differentiation of regulatory T cells, which indicates that the functional impairment of viral infected DCs orchestrates the immunosuppression in response to the acute JEV infection.

## Methods

### Reagents, virus and cells

The fluorescent antibodies, including CD11c-PE (N418), CD40-FITC (HM40-3), CD80-FITC (16-10A1), CD83-FITC (34-1-2S) and MHCⅠ-FITC (Michel-17), recombinant mouse granulocyte-macrophage colony stimulating factor (rmGM-CSF) and IL-4 (rmIL-4) were purchased from eBioscience Inc. (San Diego, CA). The anti-E (JEV envelope protein) MAb was generated in our laboratory and purified with NAb™ Spin Kits (Thermo Scietific, USA) according to the manufacturer's instructions. JEV P3 strain was produced in BHK-21 which was maintained in Dulbecco's Modified Eagle's Medium (DMEM, Sigma-Aldrich, MO) supplemented with 10% heated-inactivated fetal bovine serum (FBS, Hyclone, Logan, UT) of 100 μg/ml streptomycin and 100 U/ml penicillin (Sigma-Aldrich, MO) at 37°C with 5% CO_2_. And then the virus was tittered by plaque formation assay with BHK-21 cell line. JEV stock was treated with UV irradiation for 1 min (wavelength 253.7 nm, radiation intensity ≥ 60 μW/cm2, distance 30 cm).

### Generation of bone marrow-derived DCs (bmDCs) and spleen-derived DCs (spDCs)

For generation of bmDCs from BALB/c mouse bone marrow cultures, the procedure of Inaba et al., [1992] was used with minor modifications [47]. Briefly, the bone marrow was flushed from femurs and tibias and subsequently depleted of erythrocytes with ammonium chloride. Cells were plated at 2 × 10^6^/ml in DCs media (RPMI 1640 supplemented with 10% FBS, 100 μg/ml streptomycin, 100 U/ml penicillin, 10 ng/ml of rmGM-CSF and rmIL-4). At day 2 and 4 of culturing, 50% of the supernatant was removed and replenished with fresh DCs media. At day 6, non-adherent cells were collected and transferred into a new dish. After a total of 7 to 9 days of culturing, bmDCs were harvested and purified with StemSep™ Mouse Dendritic Cell Enrichment Kit (StemCell, Vancouver, BC, Canada).

Four-week old BALB/c mice were infected with 1 × 10^5 ^PFU of JEV P3 i.p., stimulated with identical quantity of UV-P3 or left mock-treated for 5 days. The splenocytes were obtained from P3-infected or UV-P3-stimulated or mock-treated mice. The spDCs were isolated from the splenocytes and purified with StemSep™ Mouse Dendritic Cell Enrichment Kit (StemCell, Vancouver, BC, Canada) according to the manufacturer's guidelines. The purity of the bmDCs and spDCs fraction was higher than 90% as determined by FACS analysis of CD11c. Dendritic morphology was assessed by phase-contrast microscopy and viability was assessed by trypan blue exclusion.

### JEV P3 infection of DCs

The immature bmDCs were infected with P3 at an MOI of 1. After 1 h of infection in incomplete medium (DCs media without FBS), cells were washed thoroughly three times and cultured in DCs medium. In some instances, the infected bmDCs were cultured for up to 5 days and on each day cell supernatants were collected and measured for viral RNA quantity. Similarly, the spDCs were harvested from mouse splenocytes every other day thrice after challenge with 10^5 ^PFU of JEV per mouse i.p. to detect the viral load in spDCs. Relative levels of viral load in P3-infected bmDCs or spDCs were determined by conducting quantitative real-time PCR analysis by ABI prism 7500 Sequence Detection System (Applied Biosystems) reverse transcription of total RNA isolated from infected samples. Thermal cycling conditions were 2 min at 50°C, 10 min at 94°C, 40 cycles of 15 s at 94°C and then 1 min at 60°C. Gene expression was measured by relative quantity and normalized to β-actin expression by the subtraction of Ct's to provide ΔCt values. After 3 days culture, cells were harvested and used to detect the viral production by RT-PCR and Western blotting and the samples were subjected to PCR. The consensus primers 5'-GCTCTGAAAGGCACAACC-3' (primer1) and 5'-CTGAAGGCATCACCAAAC-3' (primer2) were used to amplify the 467-bp DNA products which were specific for JEV. For Western blotting analysis, cells were collected after 3 days infection and the total proteins were separated by 10% SDS-PAGE. Separated proteins were electroblotted onto a nitrocellulose membrane. The nonspecific antibody-binding sites were blocked with 1% bovine serum albumin (BSA) in TBS-T buffer (10 mM Tris-HCl pH 8.0, 150 mM NaCl, and 0.05% Tween-20), and then membranes reacted with anti-E MAb. The resulting blot was treated with peroxidase-conjugated goat anti-mouse IgG (SouthernBiotech, USA). 3, 3-Diaminobenzidine tetrahydrochloride (DAB) was used as substrate for membrane development. The *in intro *bmDCs were harvested after 3 days infection and the *in vivo *spDCs were isolated from mice which had been challenged for 5 days. 1 × 10^5 ^bmDCs or spDCs were doubly stained with 1.0 μg FITC-anti-E and 1.0 μg PE-anti-CD11c and analyzed by FACS respectively.

### Phenotypic analysis

After 3 days *in vitro *infection or 5 days post innoculation, as described in the JEV P3 infection of DCs, the expression of maturation markers of bmDCs and spDCs were determined by FACS on a FACSCalibur (Beckton-Dickinson [BD], San Jose, CA). 1 × 10^5 ^bmDCs or spDCs were stained with surface marker antibodies including CD11c, CD40, CD80, CD83 and MHCⅠ, or isotype controls at 4°C for 30 min as per manufacturer's guidelines (eBioscience Inc., San Diego, CA). After washing three times with PBS containing 1% FBS, DCs were phenotypically analyzed by FACS.

### Analysis of cytokine production

The cytokine releases (IFN-α, TNF-α, CCL2 and IL-10) from P3-infected, UV-P3-stimulated or mock-treated bmDCs or spDCs from differently treated mice were measured by enzyme-linked immunosorbent assay (ELISA) kits (eBioscience Inc., San Diego, CA) in accordance with the manufacturer's guidelines. LPS or poly (IC) served as positive agonist. The concentrations of cytokines in the samples were accessed from the standard curves.

### T cells activation capacity of P3-infected DCs (MLR and ELISPOT assay)

Mixed lymphocyte reactions (MLR) were performed by co-incubation of 1 × 10^3^, 2 × 10^3 ^or 1 × 10^4 ^P3-infected, UV-P3-stimulated or mock-treated, bmDCs or spDCs from differently treated mice with or without 1 μg/ml LPS treatment and 1 × 10^5 ^allogeneic naive T cell per well in 96-well plates (Costar, Cambridge, MA). The mock-treated, P3-infected, UV-P3-stimulated, bmDCs and spDCs or T cells served as spontaneous NADH/NADPH release controls respectively. After 3 days of incubation in a humidified chamber at 37°C in 5% CO_2_, 50 μl of CellTiter 96^® ^AQ_ueous _One Solution Cell Proliferation Assay (Promega, Madison, WI, USA) was added to each well for 30 min at RT, and then 50 μl of stop solution (10% SDS) was added. The absorbance at 490 nm was recorded by ELISA reader (AD340; Beckman Coulter, Fullerton, CA, USA). The activities for activating T cells of differently treated bmDCs were measured as 100% (OD490_DC+T exp._-OD490_DC spont._-OD490_T spont._)/(OD490_T spont._).

P3-infected, UV-P3-stimulated or mock-treated bmDCs or spDCs from differently treated mice were harvested and treated with Mitomycin C (Sigma-Aldrich, MO) at final concentration of 10 μg/ml for 1 h and washed twice before assessment with enzyme-linked immunospot assay with Mouse IFN-γ ELISPOT Kit (eBioscience Inc., San Diego, CA). PVDF-membrane-bottomed 96-well plates (Millipore) were coated with 10 μg/ml of mAb on IFN-γ in carbonate coating buffer. The treated or mock bmDCs were seeded in triplicates (1 × 10^4 ^per well) together with 1 × 10^5 ^per well T cells. LPS (lipopolysacchide, Sigma-Aldrich, MO)-stimulated DC/T cell co-cultures were used as controls. After incubation for 20 h, cells were discarded and the plates were washed in PBS-0.05% Tween and incubated with biotinylated anti-IFN-γ mAb (1:1000). After washing, plates were incubated with HRP-Avidin, washed and incubated with AEC solution (Sigma-Aldrich, MO). The staining was stopped by rinsing with water and a red spot was counted as single spot forming unit (SFU). After rewashing, the cytokine-producing cells were visualized with substrate in accordance with the manufacturer's guidelines and counted with an automated ELISPOT reader (AID). The spot-forming T cell number was calculated as following: No._DC+T_-No._DC_.

### T cell isolation and Treg differentiation

T cells from splenocytes of BALB/c mice were enriched by StemSep™ Mouse T Cell Enrichment Kit (StemCell, Vancouver, BC, Canada) in accordance with the manufacturer's guidelines. Purified T cells were cultured in RPMI 1640 supplemented with 5% FBS, 1 × nonessential amino acids, 2 mM L-glutamine, 10 mM HEPES, 1 mM sodium pyruvate, 500 nM 2-ME, 100 μg/ml streptomycin and 100 U/ml penicillin.

To assess the impact of JEV infection on Treg cell differentiation *in vivo*, 1 × 10^5^, P3-infected, UV-P3-stimulated, LPS- or mock-treated bmDCs were added to 1 × 10^6 ^allogeneic naïve T cells in 12-well flat-bottom plates (Costar, Cambridge, MA) in triplicate. After 5 days of co-culture, *in vitro *Treg cells (CD4+ and Foxp3+) were isolated (StemCell, Vancouver, BC, Canada) and stained with Mouse Regulatory T Cell Staining Kit (eBioscience Inc., San Diego, CA) in accordance with the manufacturer's instructions and analyzed by FACS. The *in vivo *Treg in splenocytes were purified and conducted on FACS from mice challenged with 10^5 ^PFU P3 or inoculated with identical UV-P3 for 5 days or from mock-treated mice.

### Statistical analysis

Statistical analysis was performed using the Student's *t*-test. Means were considered significantly different at *P *< 0.05.

## Competing interests

The authors declare that they have no competing interests.

## Authors' contributions

SC, YL and JY carried out most of the experiments and wrote the manuscript. XY, LC and XL participated part of experiments. HC and SC conceived of the study, participated in its design and coordination, and revised the manuscript. All authors read and approved the final manuscript.
